# Hereditary diffuse gastric cancer: between underdiagnosis and overtreatment: a case series

**DOI:** 10.3389/fsurg.2026.1732442

**Published:** 2026-01-30

**Authors:** Andrea Cavallaro, Antonio Zanghì, Paolo Di Mattia, Giorgio Graziano, Filippo Sanfilippo, Luigi La Via, Alessandro Cappellani, Giorgio Giannone, Kenya Tiralongo

**Affiliations:** 1 General Surgery III, Department of General Surgery and Medical-Surgical Specialties, University of Catania, AOU Policlinico “G. Rodolico—San Marco”, Catania, Italy; 2 Department of General Surgery and Medical-Surgical Specialties, Chief ChiSMaCoTA Research Center, AOU Policlinico “G. Rodolico—San Marco”, Catania, Italy; 3 General Surgery, Umberto I Hospital, Department of Medicine and Surgery, Kore University, Enna, Italy; 4 Department of Anaesthesia and Intensive Care, AOU Policlinico “G. Rodolico—San Marco”, Catania, Italy; 5 School of Anaesthesia and Intensive Care, University of Catania, AOU Policlinico “G. Rodolico—San Marco”, Catania, Italy; 6 General Surgery Unit, Mediterranean Oncologic Institute, Catania, Italy

**Keywords:** c.1792C>T, CDH1 pathogenic variant, endoscopic surveillance, genetic counseling, hereditary diffuse gastric cancer, phenotipic penetrance, prophylactic total gastrectomy

## Abstract

**Background:**

Hereditary Diffuse Gastric Cancer (HDGC) is a rare but highly penetrant autosomal dominant cancer predisposition syndrome, most commonly associated with germline pathogenic variants in the CDH1 gene. Early diagnosis remains challenging due to the absence of specific clinical or endoscopic features in early disease stages.

**Methods:**

We present a case series describing a cluster of advanced diffuse gastric cancer (DGC) cases in a single Italian family. Clinical, genetic, and surgical data were collected and analyzed, including pedigree reconstruction, genetic testing, and risk-reducing interventions.

**Results:**

Two male siblings developed advanced signet ring cell gastric carcinoma at ages 41 and 44, both with rapid disease progression and fatal outcomes. Their family history revealed two sisters who had died from gastric cancer at a young age. Genetic counseling identified a CDH1 c.1792C>T pathogenic variant in affected family members. Two young, asymptomatic female carriers (aged 18 and 22) underwent prophylactic total gastrectomy in accordance with international guidelines. Subsequently, another male sibling died at the age of 30 due to gastric cancer. This familial cluster demonstrated high phenotypic penetrance and highlighted the impact of genetic testing on clinical management. In addition, we discuss the evolving landscape of risk stratification and the balance between prophylactic total gastrectomy and structured endoscopic surveillance.

**Conclusion:**

This case series underscores the clinical heterogeneity of HDGC and the critical role of timely genetic testing, family history assessment, and early prophylactic gastrectomy in high-risk carriers. A multidisciplinary approach integrating clinical genetics, surgery, and endoscopic expertise is essential to optimize risk-reducing strategies and outcomes in HDGC.

## Introduction

Hereditary Diffuse Gastric Cancer (HDGC) is a rare but highly penetrant autosomal dominant cancer syndrome, commonly caused by pathogenic germline variants in the CDH1 gene, which encode the epithelial cell adhesion protein E-cadherin. More rarely, pathogenic variants in the CTNNA1 gene, encoding alpha-catenin, have also been implicated.

The syndrome is primarily associated with diffuse-type gastric cancer (DGC), particularly with signet ring cell histology, and lobular breast cancer (LBC) in female carriers. Clinical presentation is often silent until advanced disease, and diffuse gastric cancer may progress rapidly despite minimal mucosal changes detectable by endoscopy, making early diagnosis extremely challenging (1, 2).

The following case series describes a cluster of advanced gastric cancer cases within the same family, which ultimately led to the identification of a germline *CDH1* mutation and subsequent prophylactic treatment in asymptomatic carriers.

## Matherial and methods

### Case 1

In June 2015, a 41-year-old male (Case 1) was admitted to the surgical department of Policlinico “G. Rodolico—San Marco” Hospital in Catania, Italy. His family history was significant for the early deaths of two sisters from gastric cancer at ages 18 and 23. The patient reported progressively worsening abdominal pain over two weeks, associated with nausea and vomiting. Abdominal ultrasound revealed ascitic fluid. Upper endoscopy demonstrated a 4 cm ulcerative lesion on the posterior wall of the gastric body. Contrast-enhanced computed tomography (CT) showed peritoneal effusion, acute gastric dilatation, and extensive circumferential thickening involving the greater curvature and antrum, along with fine peritoneal nodularity suggestive of peritoneal carcinomatosis. Histopathological examination of endoscopic biopsies confirmed gastric adenocarcinoma.

The patient underwent an *en bloc* subtotal gastrectomy, right and transverse colectomy, and hyperthermic intraperitoneal chemotherapy (HIPEC) with Mitomycin C (7.5 mg/m^2^, reduced by 25% due to borderline elevated serum creatinine).

**Figure 1:**
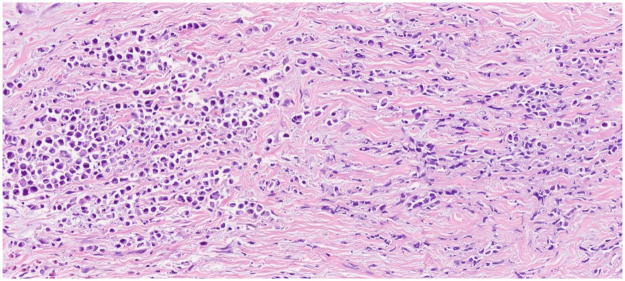
Hematoxylin and eosin poorly differentiated/signet ring cell adenocarcinoma.

Final pathological examination revealed a poorly differentiated/signet ring cell adenocarcinoma (Figure 1) infiltrating the full thickness of the gastric wall, extending into the subserosa, accompanied by serosal ulceration. Omental metastases were also identified. The tumor was pathologically staged as pT4aN1M1 according to the AJCC 7th edition criteria. Despite surgical resection and subsequent oncological treatment, the patient succumbed three weeks postoperatively due to multiple organ failure secondary to sepsis.

**Figure 2:**
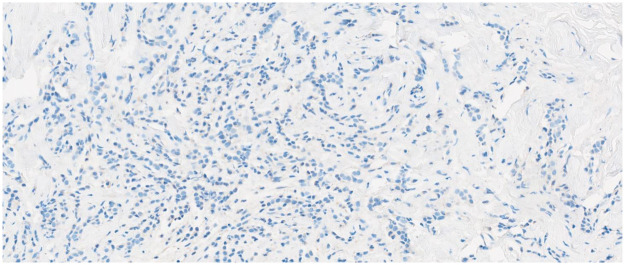
Near-complete loss of membranous E-cadherin expression: protein absent in over 90% of tumor cells.

Immunohistochemical assessment of E-cadherin expression was performed posthumously on archived formalin-fixed, paraffin-embedded tissue, with interpretation taking into account the age of the specimen. This analysis demonstrated a near-complete loss of membranous E-cadherin expression, with the protein absent in over 90% of tumor cells, consistent with the expected pathogenic effect of the germline CDH1 mutation (Figure 2).

### Case 2

In October 2017, the patient's brother (Case 2) presented to the Mediterranean Oncology Institute in Catania with persistent pyrosis. Upper gastrointestinal endoscopy revealed a large, deep ulcer along the greater curvature and diffuse thickening of the antrum, which appeared rigid and non-distensible. Biopsies confirmed poorly differentiated adenocarcinoma (CK pan-positive and CD34-negative). Contrast-enhanced CT showed marked, heterogeneous thickening of the lesser curvature, gastric body, and pyloric antrum, with increased density of surrounding mesenteric fat and multiple enlarged lymph nodes in the gastric, celiac, and mesenteric regions. The patient subsequently developed acute abdominal pain and underwent emergency laparotomy for gastric perforation at a different hospital with an emergency department.

A gastric resection with Roux en Y gastro jejunal anastomosis was performed. Immunohistochemical analysis on the specimen showed retained expression of all MMR proteins (MLH1, MSH2, MSH6, PMS2), consistent with microsatellite stability. Postoperatively, he received adjuvant chemotherapy with the FLOT regimen.

In February 2018, the patient underwent a second laparotomy for intestinal obstruction at the Mediterranean Oncology Institute in Catania. Intraoperatively, the supra mesocolic space was completely replaced by a massive omental cake, with extensive involvement of the transverse mesocolon and both the afferent and efferent limbs of the previous anastomosis. Surgical management included an ileo-ileal anastomosis and a repeat gastro jejunal anastomosis.

The patient died a few days later due to postoperative complications and sepsis.

### Familiar genetic counseling and molecular findings

In light of the aggressive and early-onset nature of gastric cancer in multiple family members, genetic counseling was recommended. The mother of the two male patients had previously undergone surgery for breast cancer, and her brother had died of gastric cancer further suggesting a hereditary cancer syndrome. Next-generation sequencing of the *CDH1* and CTNNA1 genes was performed on the surviving brothers and on the daughters of the deceased patients. A heterozygous pathogenic variant, c.1792C>T, was identified.

No pathogenic variants were detected in MLH1, MSH2, MSH6, PMS2, EPCAM**,** APC, MUTYH, BRCA1 or BRCA2 all of which were included in the hereditary cancer gene panel.

This variant was detected in:

- One of the surviving brothers (Case 3)
- The daughter of one of the deceased sisters
- The daughter of patient 1

**Figure 3:**
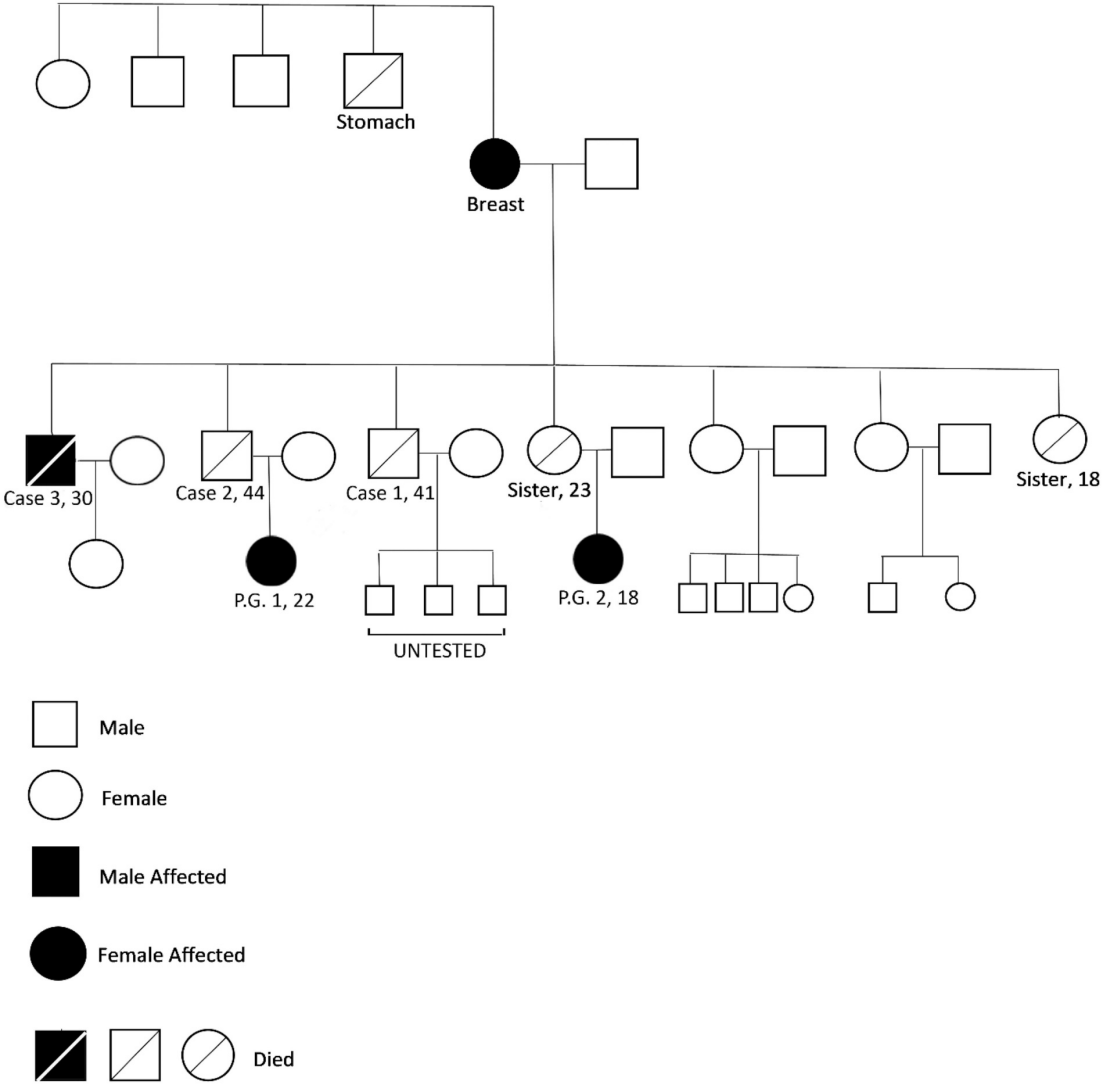
A family pedigree was obtained during the genetic counseling session.

The other two sisters tested negative for the variant, while several other family members have not yet undergone genetic testing (Figure 3).

The c.1792C>T (p.Arg598*) variant introduces a premature stop codon, which is expected to produce a truncated, non-functional CDH1 protein. According to the American College of Medical Genetics and Genomics (ACMG) and Association for Molecular Pathology (AMP) guidelines, this type of loss-of-function alteration represents a well-established pathogenic mechanism for HGDC (PVS1) and occurs at a residue where other pathogenic truncating variants have been described (PM5_Supporting). The variant is considered rare (PM2_Supporting) and a confirmed *de novo* occurrence has been documented (PS2). Robust case level evidence (3–6) from multiple unrelated affected families (PS4_strong) demonstrates that the variant co-segregates with hereditary diffuse gastric cancer across more than seven meioses, providing strong support for pathogenicity (PP1_Strong). These criteria justify classifying c.1792C>T (p.Arg598*) as a pathogenic variant in FDA-recognized human genetic variant databases.

### Prophylactic surgery

**Figure 4:**
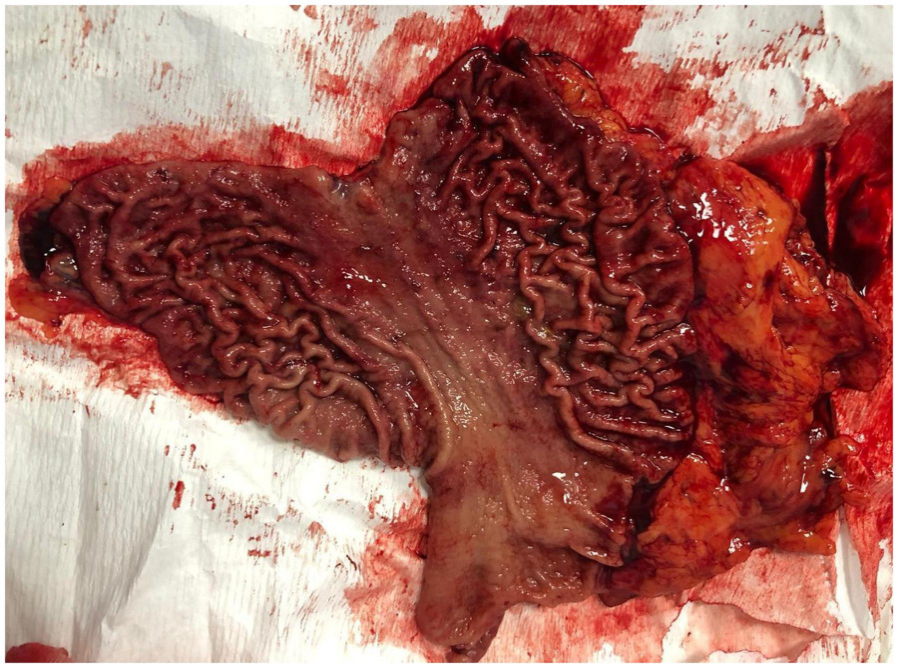
Prophylactic total gastrectomy with section of the stomach 3 cm above the cardia.

**Figure 5:**
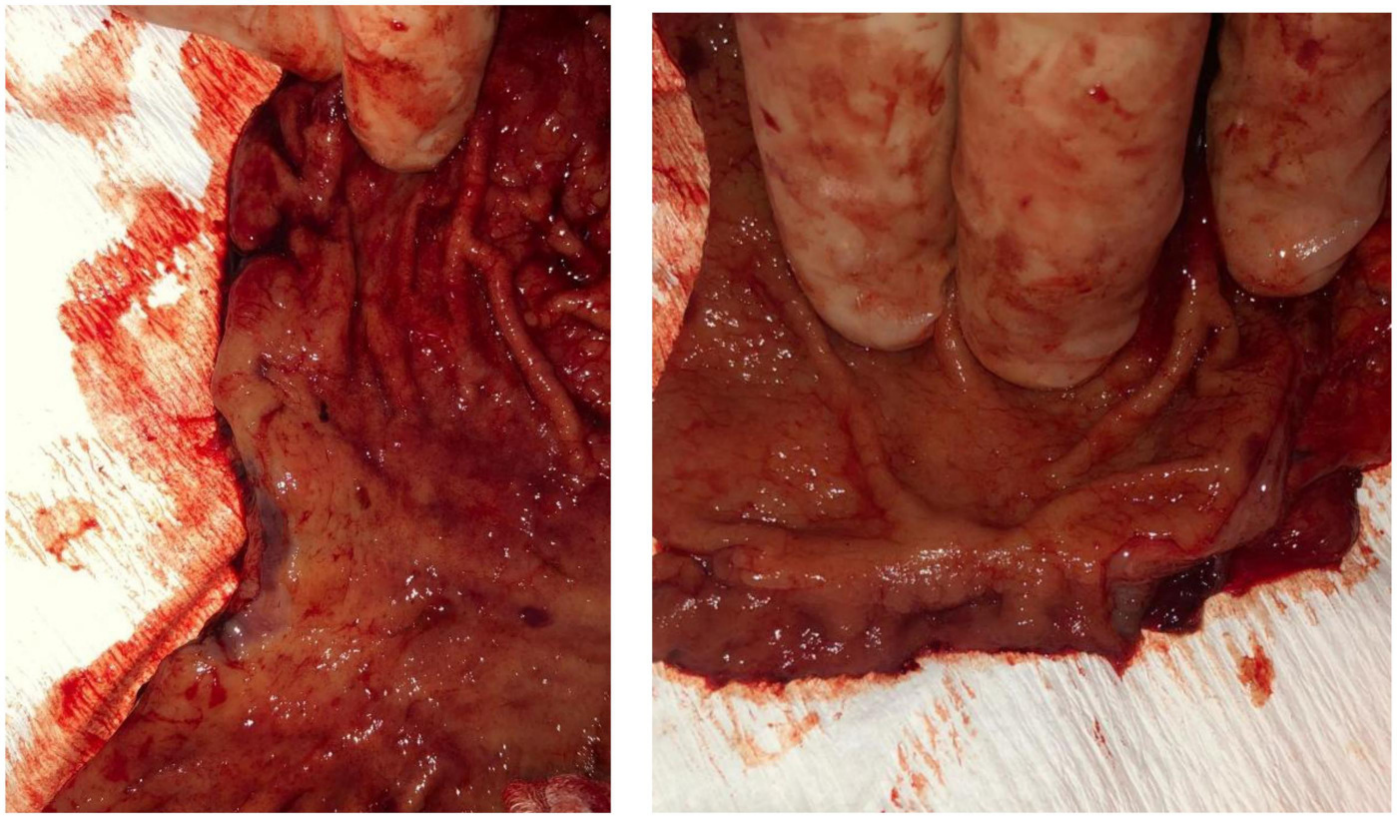
Prophylactic total gastrectomy with section of the stomach 3 cm above the first cm of the duodenum.

Despite their young age (22 and 18 years), the two identified female carriers underwent prophylactic total gastrectomy via a conventional open approach, with resection performed 3 cm above the cardia and including the first centimeter of the duodenum (Figures 4 and 5).

In the older patient, the surgical specimen consisted of a total gastrectomy measuring 13 cm along the lesser curvature and 19 cm along the greater curvature, with a 2.5 cm esophageal cuff. Histological examination revealed chronic active, micro-erosive gastritis, accompanied by edema and vascular congestion. No significant atypia or foci of invasive carcinoma were identified in either the mucosa or submucosa. All locoregional lymph nodes were free of metastasis, and surgical margins were clear.

In the younger patient**,** the total gastrectomy measured 13 cm along the lesser curvature and 20 cm along the greater curvature, with a 3.5 cm esophageal cuff. Microscopic evaluation showed superficial chronic gastritis with edema and vascular congestion. Helicobacter pylori testing was negative. Surgical margins and esophageal cuff were uninvolved.

This preventive approach aligns with current recommendations for pathogenic CDH1 mutation carriers, given the high lifetime risk of diffuse gastric cancer, the challenges in early detection, and the occurrence of multiple deaths likely related to the syndrome within the family.

### Case 3

The third patient tested, a 30-year-old man, although fully informed about his status as a carrier of a CDH1 gene mutation and the associated risks, showed limited willingness to undergo prophylactic surgery or to adhere to endoscopic surveillance. Some symptoms appeared during 2020, while the patient was in detention. He subsequently underwent esophagogastroduodenoscopy, which revealed an extensive ulcerative lesion along the lesser curvature. Following staging and multidisciplinary oncologic team evaluation, the patient completed four cycles of neoadjuvant FOLFOX, ending in February 2021.

In March 2021, he underwent partial gastrectomy with D2 lymphadenectomy at the Mediterranean Oncology Institute in Catania. The resected specimen consisted of a partial gastrectomy measuring 17 × 6 cm, containing a 9-cm ulcerated and vegetating lesion, located 2 cm from the distal resection margin.

Additionally, a 6-cm segment of transverse colon with opaque serosa was included. The gastric lesion extended through the entire thickness of the gastric wall and perforated the serosa. The diagnosis was high-grade, poorly differentiated, poorly cohesive gastric adenocarcinoma (WHO classification), of the diffuse/signet-ring cell type (Lauren classification). The tumor also infiltrated the perivisceral adipose tissue of the transverse colon.

There was extensive lymphovascular, venous, and perineural invasion. Metastases were found in 13 of 16 nodes from the lesser curvature, 9 of 26 nodes from the greater curvature, and 1 additional node, for a total of 23 metastatic lymph nodes. The surgical resection margins were free of tumor. The transverse colon segment showed diffuse infiltration by high-grade gastric adenocarcinoma involving the perivisceral adipose tissue. Peritoneal cytology revealed red blood cells and inflammatory cells, with no malignant cells reported. The pathologic stage was ypT4b N3b according to the AJCC 8th edition. Tumor regression grade (TRG) was 3, indicating a poor response to FOLFOX chemotherapy with extensive residual tumor. HER2 status was negative (score 0), assessed using the Ventana Benchmark Ultra platform with the PATHWAY anti-HER2/neu (4B5) antibody. Mismatch repair (MMR) immunohistochemistry demonstrated preserved nuclear expression of MLH1, MSH2, MSH6, and PMS2 (clones M1, G219-1129, SP92, and A16-4; Roche), performed on the Ventana platform, consistent with a mismatch repair–proficient (pMMR) profile. The patient died several months later.

## Discussion

Hereditary Diffuse Gastric Cancer (HDGC) is an autosomal dominant cancer predisposition syndrome associated with an elevated lifetime risk of diffuse gastric cancer (DGC) and lobular breast carcinoma (LBC). Germline heterozygous pathogenic variants (PVs) in the CDH1 gene represent the primary genetic cause of HDGC, with an estimated prevalence of 1 in 5,000 to 1 in 8,000 in unselected populations (1).

The CDH1 gene encodes E-cadherin, a transmembrane glycoprotein critical for epithelial cell–cell adhesion and the maintenance of tissue architecture. Tumorigenesis in CDH1 carriers typically follows the two-hit model, in which somatic inactivation of the wild-type allele leads to complete loss of E-cadherin expression and drives neoplastic transformation (2, 7).

Truncating mutations in the *CTNNA1* gene have also been identified as contributors to HDGC predisposition. Carriers of these variants show loss of *α*E-catenin expression, establishing *CTNNA1* as a moderate-penetrance HDGC gene with demonstrated associations with DGC and LBC (8).

Alterations in key molecular pathways, including β-catenin–driven Wnt signaling, epithelial-to-mesenchymal transition (EMT), and E-cadherin–mediated cell adhesion, play a pivotal role in gastric cancer development. β-catenin overexpression (membranous, cytoplasmic, or nuclear) is significantly associated with tumor differentiation, whereas progressive loss of E-cadherin expression correlates with poorly cohesive and diffuse histologic subtypes, poor differentiation, and recurrence, and shows a trend toward reduced survival (9).

Histopathological studies indicate that the vast majority of *CDH1* mutation carriers (88%–97%) harbor multiple microscopic foci (0.1–10 mm) of intramucosal signet ring cell carcinoma (SRCC), confined to the superficial mucosa and classified as pT1a lesions. The natural history and risk of progression of these early lesions to invasive or advanced-stage DGC (>pT1a) remain poorly defined (1, 2); most prophylactic total gastrectomy (PTG) specimens show only intramucosal SRCC (pT1a), whereas pT1b or more advanced lesions are observed in approximately 2%–3% of cases (1, 2, 7).

Historically, *CDH1* mutation carriers were believed to have a lifetime gastric cancer risk of 25%–80%, leading to the widespread recommendation of PTG, particularly in young, asymptomatic individuals or those with a positive family history. However, emerging evidence indicates a more heterogeneous (Table 1) risk profile (10–12).

**Table 1:** Cumulative risk according to NCCN 2025 guidelines.

Cancer type	Average age of presentation	Cumulative risk for diagnosis through age 80 years	Cumulative risk for diagnosis through lifetime for general population	References:
Stomach (Diffuse)	47–49 years	24.7%–33% Females 37.2%–42% Males including Stage I. (Stage II or more cancer risk: 10% males/7% females)	0.8%	(10, 11, 13)
Breast (Lobular)	51–54 years	37%–55% females	12.9% females	(10–12)

A pivotal 2024 multicenter cohort study including 213 North American families (7,323 individuals) carrying pathogenic or likely pathogenic *CDH1* variants reported lower overall penetrance than previously assumed. The cumulative risk of advanced gastric cancer by age 80 was 10.3% in males and 6.5% in females, while the lifetime risk of lobular breast cancer in females reached 37.2% (13). Importantly, family history remains a strong risk modifier: carriers with three first-degree relatives affected by gastric cancer had an estimated lifetime risk of 38% (13).

These findings have prompted a paradigm shift in clinical guidelines. The International Gastric Cancer Linkage Consortium (IGCLC) recommends a more individualized approach to risk stratification and surgical timing, taking into account mutation type, family history, and patient preferences (14).

Updated criteria for *CDH1* testing have expanded to include patients with isolated lobular breast cancer (LBC), bilateral breast cancer, or signet ring cell carcinoma, even in the absence of a strong family history (15):

1. Individuals with DGC at any age
2. Families with ≥2 cases of gastric cancer, with at least one diagnosed at age ≤50 years or confirmed DGC at any age
3. Individuals meeting NCCN testing criteria for high-penetrance breast cancer susceptibility genes

Management strategies for CDH1 PV carriers include prophylactic gastrectomy or structured endoscopic surveillance. Despite advances in endoscopy, including chromoendoscopy and magnification techniques, endoscopic surveillance remains suboptimal: random biopsies frequently miss occult SRCC, and superficial samples often cannot distinguish pT1a from pT1b lesions (16–18).

Gastrectomy with D1 or D2 lymphadenectomy is generally indicated for patients with confirmed pT1b or higher lesions or persistent symptoms suggestive of advanced disease.

Prophylactic total gastrectomy with Roux-en-Y reconstruction has traditionally been the recommended treatment for CDH1 PV carriers with a family history of gastric cancer, although it is associated with significant postoperative morbidity and long-term sequelae.

More recently, endoscopic surveillance has emerged as a potential alternative to prophylactic gastrectomy for individuals carrying germline *CDH1* pathogenic variants, although current data are limited by short follow-up periods. Long-term outcomes of surveillance remain poorly defined, particularly regarding progression to advanced disease and disease-specific mortality. To date, no gastric cancer–related deaths have been reported among surveillance cohorts; however, studies are constrained by brief follow-up and a high rate of conversion to surgery, often without prior endoscopic detection of SRCC (19–22).

Asif et al. reported outcomes from 270 *CDH1* carriers, demonstrating that endoscopic surveillance can be a viable alternative to prophylactic total gastrectomy (PTG) for selected individuals who decline surgery. The low incidence of advanced gastric cancer (stage > T1a) suggests that, in carefully selected patients, surveillance may constitute a reasonable and clinically appropriate management strategy (23).

Surveillance should be performed in expert centers and should include both targeted and random biopsies. The recommended number of random biopsies is 28–30, distributed as follows: 3–5 from the cardia, 5 from the fundus, 10 from the body, 5 from the transition zone, and 5 from the antrum. Gastric inlet patches in the esophagus should be documented, inspected, and biopsied. Endoscopic features that may suggest more advanced disease, even in the absence of histologic confirmation, include reduced gastric distensibility, deep ulcerations, rigid or thickened folds, disordered vascular patterns, disrupted pit patterns, and diffuse mucosal irregularities (14, 15, 23, 24).

For carriers without evidence of advanced disease, shared decision-making is essential. Patients should be counseled on the relative benefits and limitations of risk-reducing surgery vs. structured endoscopic surveillance, taking into account the advantages and drawbacks of each strategy alongside individual values and preferences. Ideally, this process occurs within a multidisciplinary team including clinical genetics, advanced endoscopy, surgical oncology, medical oncology, and psychosocial support. Surgical timing, particularly in adolescents and younger patients with complex family histories, should be individualized, considering family history, age of onset among relatives, and psychological readiness (22, 25).

Prophylactic total gastrectomy (PTG) remains the intervention offering maximal risk reduction and is often preferred by individuals with a strong familial cancer history or high personal risk perception. Decisions to proceed with surgery are frequently influenced by personal or familial experiences with gastric cancer, especially cases involving early-onset or fatal outcomes. Conversely, some patients may favor surveillance to avoid surgical morbidity, nutritional consequences, and quality-of-life impact. However, surveillance carries the risk of false negatives and delayed diagnosis, and current evidence regarding its efficacy in preventing advanced disease or reducing gastric cancer–specific mortality remains limited.

According to the updated IGCLC guidelines, PTG continues to be the standard of care for *CDH1* PV carriers.

In this context, robotic surgery appears to be a viable option for the treatment of gastric neoplastic disease, allowing precise dissection and adequate lymphadenectomy even in anatomically complex areas (26).

Nonetheless, there is growing recognition that surveillance in expert centers can be offered to patients who wish to defer surgery or whose risk remains uncertain (14–17). Endoscopic monitoring may be considered instead of PTG depending on the individual's risk profile and personal preferences. This approach is particularly relevant for pathogenic variant carriers with uncertain DGC risk, such as those who do not meet HDGC genetic testing criteria or carry CTNNA1 pathogenic variants.

This case series describes a cluster of advanced diffuse gastric cancer (DGC) cases in a single Italian family carrying the germline CDH1 c.1792C>T pathogenic variant, which exhibits high penetrance, and reports multiple deaths likely attributable to the syndrome within the family. The c.1792C>T (p.Arg598*) variant introduces a premature stop codon, leading to a truncated, non-functional CDH1 protein. According to the ACMG and AMP guidelines, this loss-of-function mechanism is well-established for hereditary diffuse gastric cancer (HGDC) (PVS1), occurs at a residue with previously reported pathogenic truncating variants (PM5_Supporting), is rare (PM2_Supporting), and has been observed *de novo* (PS2). Strong evidence from multiple unrelated families (PS4_Strong) shows co-segregation with disease over more than seven meioses (PP1_Strong), supporting its classification as a pathogenic variant in FDA-recognized genetic databases (3–6).

This highlights the broad clinical spectrum of HDGC and emphasizes the importance of a detailed family history, timely genetic testing, evaluation of the specific mutation and any coexisting mutations, genetic counseling, shared decision-making, and early risk-reducing surgery to optimize patient outcomes.

## Conclusion

Managing HDGC is challenging, given the variable penetrance of CDH1 pathogenic variants, the limitations of current surveillance strategies, and the considerable implications of prophylactic total gastrectomy. Early recognition of hereditary cancer syndromes such as HDGC, in combination with genetic counseling, testing, and individualized risk-reducing strategies, can significantly influence the natural history of the disease. Optimal management relies on a multidisciplinary approach, including oncologists, geneticists, surgeons, pathologists, and psychosocial support teams, to ensure that clinical decisions are guided both by medical evidence and by patient values and preferences (27–30).

## Data Availability

The original contributions presented in the study are included in the article/Supplementary Material, further inquiries can be directed to the corresponding author.
